# Manipulation of Bryophyte Hosts by Pathogenic and Symbiotic Microbes

**DOI:** 10.1093/pcp/pcx182

**Published:** 2017-11-21

**Authors:** Philip Carella, Sebastian Schornack

**Affiliations:** Sainsbury Laboratory, University of Cambridge, 47 Bateman Street, Cambridge, UK

**Keywords:** Bryophytes, Fungi, Hornworts, Liverworts, Plant–pathogen interactions, Symbiosis

## Abstract

The colonization of plant tissues by pathogenic and symbiotic microbes is associated with a strong and directed effort to reprogram host cells in order to permit, promote and sustain microbial growth. In response to colonization, hosts accommodate or sequester invading microbes by activating a set of complex regulatory programs that initiate symbioses or bolster defenses. Extensive research has elucidated a suite of molecular and physiological responses occurring in plant hosts and their microbial partners; however, this information is mostly limited to model systems representing evolutionarily young plant lineages such as angiosperms. The extent to which these processes are conserved across land plants is therefore poorly understood. In this review, we outline key aspects of host reprogramming that occur during plant–microbe interactions in early diverging land plants belonging to the bryophytes (liverworts, hornworts and mosses). We discuss how further knowledge of bryophyte–microbe interactions will advance our understanding of how plants and microbes co-operated and clashed during the conquest of land.

## Introduction

Interactions between plants and microbes can have beneficial, neutral or detrimental effects on host fitness. In general, most plants are resistant to most microbes, as they employ several layers of sophisticated immune mechanisms to ward off intruders (Jones and Dangl 2006). However, adapted microbes have co-evolved to circumvent and/or suppress immunity in order to colonize plant tissues. This strategy is employed by microbes with biotrophic or hemi-biotrophic lifestyles that require a living host to supply nutrients. Such microbes include symbiotic fungi and bacteria, which enter into mutually beneficial interactions for the exchange of resources, and biotrophic pathogens that manipulate plant cells to obtain nutrients without providing any in return. Microbes that have a necrotrophic lifestyle actively kill host tissues and metabolize the remains, which is thought to be a more generalist approach.

Much of what we know about plant–microbe interactions is derived from studies performed in angiosperms, which have revealed conserved and unique mechanisms across a diverse range of flowering plants. From the microbial perspective, angiosperm hosts present several similarities, including modes of reproduction, resource acquisition and allocation, tissue development and architecture, hormone signaling and cell wall composition. It is therefore reasonable to suspect that microbes employ similar mechanisms for the manipulation of different angiosperm hosts. However, plant–microbe interactions are present throughout the plant kingdom and are represented in the fossil record (Martin et al. 2017), which demonstrates that microbes have the capacity to colonize a more diverse set of plants than are currently investigated. This includes plants belonging to the paraphyletic bryophyte group, which represent the closest living relatives of the first land plants (Renzaglia et al. 2007, Ligrone et al. 2012). Bryophytes are non-vascular, gametophyte-dominant (haploid) plants that include liverworts, hornworts and mosses. Several interactions with pathogenic or symbiotic microbes have been described in bryophytes, with recent interest in establishing new pathosystems to study the evolution of plant–microbe responses (Ponce de Leon and Montesano 2017). Such studies are beginning to provide key information on conserved and novel measures that either permit or prevent the colonization of bryophyte tissues by microbes.

## Microbial Colonization of Bryophyte Tissues and Cells

**Fig. 1:**
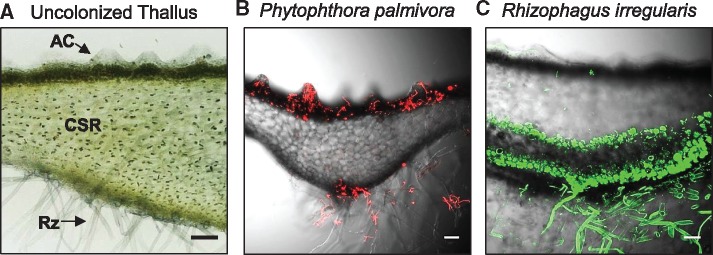
Differential colonization of the liverwort thallus by pathogenic and symbiotic microbes. (A) Light micrograph of a sectioned liverwort thallus (*Lunularia cruciata*) demonstrating air chambers (AC) on the dorsal photosynthetic layer, the central storage region (CSR) and rhizoids (Rz) emanating from the ventral epidermis. (B) Colonization of *L. cruciata* thalli by the hemi-biotrophic oomycete pathogen *Phytophthora palmivora* (Accession P3914) constitutively expressing tdTomato. Confocal fluorescence microscopy of sectioned thalli of 2-month-old *L. cruciata* plants 7 d after infection. Merged *z*-stack images display red fluorescence and bright field images. Plants were grown under a short-day photoperiod (50 μE m^−2^ s^−1^ light levels) on mycorrhization (M) media. (C) Colonization of *L. cruciata* thalli by the symbiotic fungus *Rhizophagus irregularis.* Confocal fluorescence microscopy of sectioned thalli stained with WGA (wheat germ agglutinin)–Alexa488 to detect fungal chitin. Plants were grown on vermiculite supplemented with crude *R. irregularis* inoculum for 2 months under a long-day photoperiod (16 h light) with attenuated light (approximately 50–70 μE m^−2^ s^−1^). Merged *z*-stack images display green fluorescence and bright field images. Scale bars = 100 μm.

Bryophytes are non-vascular plants with leafy-like or thalloid body plans. Leafy bryophytes include mosses and some liverwort species (*Jungermanniaceae*) which develop photosynthetically active leaf-like appendages that emanate from a stem (or gametophore) in addition to a below-ground axis with root hair-like rhizoids that acquire nutrients and water from the substrate (Renzaglia et al. 2007, Ligrone et al. 2012). In contrast, thalloid liverworts and hornworts display a compact, lobed morphology that constitutes the entirety of the plant body. The thallus generally contains a photosynthetically active cell layer on the dorsal surface, a central non-photosynthetic storage region and a ventral epidermal layer from which rhizoids and other structures develop (an example of a sectioned liverwort thallusis is shown in Fig. 1A). Certain liverworts, including the model *Marchantia polymorpha*, develop complex air chambers on the dorsal photosynthetic layer. These structures provide intercellular spaces that enable gas exchange between photosynthetic filament cells and the open environment via a constitutively open pore (Shimamura 2016, Villarreal et al. 2016). Below, we summarize key aspects of microbial colonization observed in the different tissues of bryophytes.

Symbiotic endophytes have been observed in each of the three bryophyte clades (liverworts, hornworts and mosses). Bryophyte–bacterial symbioses are largely represented by interactions with nitrogen-fixing cyanobacteria that are most prevalent in hornworts. Colonies of the cyanobacterium *Nostoc punctiforme* are present inside slime cavities within hornwort thalli, in symbiotic auricle structures (cavities) located on the ventral surface of certain liverworts (*Blasia* and *Caviculara*), and are also observed growing endo- or epiphytically on moss leaves (Adams and Duggan 2008). Interactions with symbiotic arbuscular mycorrhizal (AM) fungi belonging to the Glomeromycotean group are also observed in bryophytes, but are best described in liverworts (Pressel et al. 2010). Several liverwort species are colonized by AM fungi, which penetrate and grow through rhizoids in order to colonize the non-photosynthetic storage region of liverwort thalli where fungal structures such as vesicles (storage) and arbuscules (resource exchange) are produced (Ligrone et al. 2007). The colonization of liverwort thalli by AM fungi improves plant fitness, which is accelerated under simulated palaeozoic levels of CO_2_ (Humphreys et al. 2010). Notably, interactions with AM fungi display *Paris*-type morphology, with prolific intracellular hyphal growth within and between non-photosynthetic, parenchymatous cells of the liverwort thallus (Ligrone et al. 2007, Pressel et al. 2010). Glomeromycotean fungi also colonize hornworts, producing intracellular hyphae, arbuscules and vesicles; however, access to thalli is believed to occur via slime cavities rather than rhizoids (Schussler 2000, Desiro et al. 2013). Mosses also support endophytic growth of AM fungi, displaying fungal vesicles, intracellular hyphae and intracellular hyphal coils; however, there is no evidence to suggest that a functional symbiosis is established (Zhang and Guo 2007, Pressel et al. 2010). Bryophytes also engage in beneficial endophytic interactions with members of the mucoromycotina, ascomycota and basidiomycota groups. Both liverworts and hornworts interact with endogone fungi of the mucoromycotina group, which grow inter- and intracellularly to produce intracellular hyphal coils and fungal lumps within densely packed cells of the central storage region (Desiro et al. 2013, Field et al. 2015, Field et al. 2016). Ascomycetes and basidiomycetes are observed within rhizoids as well as the central storage region of liverwort thalli, and are believed to impart beneficial impacts on plant health (Duckett et al. 2006, Pressel et al. 2010).

Several studies have described plant–pathogen interactions in the model mosses *Physcomitrella patens* and *Funaria hygrometrica.* Most of these studies describe interactions with necrotrophic fungi (*Alternaria*, *Atrididymella*, *Botrytis* and *Fusarium*), oomycetes (*Pythium*) or bacteria (*Pectobacterium*), which colonize and degrade the leaves, stems and rhizoids of mosses (Davey et al. 2009, Lehtonen et al. 2009, Ponce de Leon 2011, Lehtonen et al. 2012, Ponce de Leon and Montesano 2013, Mittag et al. 2015). Classically described biotrophic or hemi-biotrophic pathogens also colonize moss tissues, with studies demonstrating intracellular hyphal growth of *Phytophthora* (*P. infestans* and *P. capsici*) and *Colletotrichum gloeosporioides* in protonemal (young differentiating gametophytes) or leaf cells of *P. patens* (Reboledo et al. 2015, Overdijk et al. 2016). In contrast, information regarding pathogenic interactions in hornworts and liverworts is extremely limited. Pathogenic oomycetes of the *Pythium* genus were isolated from mats of naturally growing leafy liverworts (*Cephaloziella varians*) in Antarctica alongside additional soil-borne fungi (*Geomyces pannorum*, *Phoma herbarum*, *Mortierella parvisporum* and *Verticillium* sp.) that could potentially affect plant health (Hughes et al. 2003). In addition, a differential capacity for powdery mildew spore establishment on *M. polymorpha* surfaces has been described, such that *Oidium neolycopersici* forms fungal penetration structures (appressoria) on liverwort thalli while *Erysiphe trifoliorum* structures are destroyed (Takikawa et al. 2014). Recently, we determined that the hemi-biotrophic oomycete pathogen *Phytophthora palmivora* colonizes the photosynthetic layer of liverworts and develops digit-like as well as highly branched haustoria-like structures to establish a biotrophic interface (Carella et al. 2017; see Fig. 1B). Additional efforts are beginning to describe bacterial species that naturally associate with liverworts (Tang et al. 2016, Alcaraz et al. 2017); however, functional data demonstrating pathogenic interactions remain to be described. Further efforts are therefore required to identify and characterize pathogenic interactions in liverworts and hornworts.

To contextualize the liverwort tissues/layers discussed above, the differential colonization of *Lunularia cruciata* thalli by the oomycete pathogen *P. palmivora* or the symbiotic arbuscular mycorrhizal fungus *Rhizophagus irregularis* is displayed in Fig 1B and C. *Phytophthora palmivora* hyphae (red) colonize air chambers of the dorsal photosynthetic layer and are sometimes associated with ventral epidermal cells and rhizoids, but not within the central storage region (Fig. 1B). In comparison, hyphae of the symbiotic fungus *R. irregularis* (green) are observed in rhizoids and within the central storage region of the thallus where arbuscules are formed (Fig. 1C). Rather than classifying microbial colonization as root or foliar, we suggest a more meaningful interpretation to be the colonization of photosynthetic or non-photosynthetic tissues. We refrain from considering the entire thallus itself as a photosynthetic unit, which others have described when referring to a unique mycorrhizal colonization of photosynthetic liverwort thalli.

## Reprogramming of Bryophyte Host Cells Induced by Microbes

The colonization of plant tissues is typically associated with host cell reprogramming, which includes changes in cell wall composition, subcellular reorganization, activation of hormones and microbe-associated signaling pathways, changes in host gene expression and the direct actions of microbial compounds and/or proteins that subvert the host cell machinery. These phenomena are well described in vascular plants; however, the degree to which they are conserved during bryophyte–microbe interactions is still unclear. Recent developments in each of these aspects of host reprogramming that occur during the colonization of bryophytes by symbiotic or pathogenic microbes are discussed below.

### Changes in cell wall composition and subcellular dynamics

The reinforcement of host cell walls is a highly conserved strategy for the containment of invading microbes. Deposits of cell wall materials at host–microbe contact sites are commonly observed in vascular plants and are even present in 400 million-year-old fossils of ancient plant cells infected with certain fungi (Krings et al. 2007). Bryophytes similarly employ this strategy. The mosses *P. patens* and *F.hygrometrica* accumulate callose, a β1-3 glucan associated with cell wall strengthening, and other host cell wall materials around pegs of invading fungal and oomycete hyphae (Martinez-Abaigar et al. 2005, Davey et al. 2009, Oliver et al. 2009, Davey et al. 2010, Lehtonen et al. 2012, Reboledo et al. 2015, Bressendorff et al. 2016, Yan et al. 2017). In addition, moss infected with *C. gloeosporioides* incorporate de novo synthesized phenolic compounds into infected cell walls, which is believed to enhance structural integrity (Reboledo et al. 2015). In some cases, the deposition of cell wall materials at contact sites, or the encasement of intracellular hyphae, is sufficient to prevent infection (Davey et al. 2010, Lehtonen et al. 2012, Overdijk et al. 2016). However, cell wall reinforcement does not always prevent pathogens from entering plant cells, as this response is observed during several interactions with disease-causing microbes (Martinez-Abaigar et al. 2005, Davey et al. 2009, Reboledo et al. 2015, Yan et al. 2017). This has also been observed in liverworts, where the colonization of *M. polymorpha* thalli by *P. palmivora* induces the accumulation of callose that is not sufficient to prevent intracellular colonization (Carella et al. 2017). Together, these studies demonstrate that cell wall reinforcement is an ancient response to colonization that is not always capable of limiting pathogen ingress.

Colonization of host tissues by symbiotic microbes is also associated with changes in cell wall composition; however, these must be permissive rather than preventive in order to establish intracellular symbioses. This may be achieved by the action of microbially derived cell wall-modifying enzymes such as cellulases/endoglucanases, although a suite of colonization-induced host proteins appear to play a more significant role (Balestrini and Bonfante 2014, Rich et al. 2014). For example, host-encoded wall-loosening expansin proteins are induced during mycorrhizal and rhizobial symbioses and are believed to contribute non-enzymatically to the local softening of plant cell walls (Cosgrove 2000, Giordano and Hirsch 2004, Balestrini et al. 2005, Dermatsev et al. 2010). The idea of host-driven cell wall remodeling is further supported by the marked lack of cell wall-degrading enzymes encoded in the genome of the AM fungus *R. irregularis* (Tisserant et al. 2013). In bryophytes, the action of cell wall-modifying proteins during symbioses is not clear; however, some wall-associated phenotypes have been described. In marchantialean liverworts, colonization by AM fungi suppresses the autofluorescent properties of non-photosynthetic cells within the thallus storage region, which is hypothesized to signify changes in phenolic compounds within the cell wall (Ligrone et al. 2007). Moreover, callose deposition is not observed at fungal penetration sites or at intracellular endosymbiotic structures, whereas antibodies recognizing fucosylated side groups associated with xyloglucan and rhamnogalacturonan host cell wall components (CCRC-M1) strongly label fungal-colonized cell walls and host–fungus interfaces (Ligrone et al. 2007). Together, these data demonstrate that similarly to vascular plants (Ballestrini et al. 1996), liverworts permit invading symbiotic fungi access to their cells and generate de novo synthesized host membranes for the accommodation of symbiotic fungi.

Subcellular reorganization is often associated with the colonization of angiosperms by filamentous microbes (Hardham et al. 2008, Pumplin and Harrison 2009). In bryophytes, several changes in host subcellular organization are observed during interactions with symbiotic or pathogenic microbes. In moss, filamentous necrotrophic pathogens induce cytoplasmic shrinkage as well as chloroplast repositioning and degradation, which ultimately leads to cell death (Davey et al. 2009, Oliver et al. 2009, Ponce de Leon 2011, Lehtonen et al. 2012, Winter et al. 2014, Yan et al. 2017). In comparison, interactions between *P. patens* and the hemi-biotrophic pathogen *P. capsici* are associated with the accumulation of cytoplasm and the repositioning of actin filaments and nuclei towards sites of hyphal invasion (Overdijk et al. 2016). Similar responses are observed during the colonization of liverwort and hornwort cells by symbiotic fungi, where fungal invasion is associated with the accumulation of active cytoplasm, repositioning of mitochondria and nuclei, fragmentation of the central vacuole and reduced chloroplast starch content (Schussler 2000, Carafa et al. 2003, Ligrone et al. 2007, Field et al. 2016). The conservation of these responses in early divergent land plants demonstrates that host subcellular reorganization is a crucial aspect of microbial colonization.

### Activation of microbe-associated signaling pathways

The perception of microbially derived stimuli is integrated in complex regulatory networks that control symbiosis and defense programs in vascular plants, of which the detection of microbial motifs in the extracellular space represents a crucial first step (Zipfel and Oldroyd 2017). Plants often perceive common microbial motifs, termed microbe-associated molecular patterns (MAMPs), through the extracellular domains of pattern recognition receptor (PRR) proteins (Boller and Felix 2009). The detection of MAMPs subsequently activates intracellular kinase domains that initiate mitogen-activated protein (MAP) kinase signaling cascades, ultimately activating basal defense responses such as defense gene expression, cell wall reinforcement and the accumulation of reactive oxygen species (ROS) and defense hormones (Boller and Felix 2009, Meng and Zhang 2013). Symbiosis programs similarly require the detection of microbial motifs; however, they are often modified from immunogenic counterparts. For example, symbiotic microbes add lipid moieties to *N*-acetylglucosamine-containing molecules to differentiate from immunogenic MAMPs such as fungal chitin and bacterial peptidoglycan (Zipfel and Oldroyd 2017).

Significant progress has been made in deciphering MAMP perception in the model moss *P. patens*, which perceives fungal chitin using a conserved CERK1 receptor that subsequently activates the MPK4a/b kinases, leading to growth inhibition, defense gene expression and cell wall reinforcement (Bressendorff et al. 2016). Additional aspects of the MAMP-triggered immunity (MTI) response appear to be conserved in bryophytes, based on observations that moss infected with fungi and oomycetes accumulate ROS species such as hydrogen peroxide and superoxide anions, up-regulate the *PAL* (*Phenylalanine Ammonia-Lyase*) and *CHS* (*Chalcone Synthase*) defense genes, and undergo cell wall reinforcement as described above (Ponce de Leon and Montesano 2017). Mosses also accumulate defense hormones and their precursors during infection. The defense hormone salicylic acid (SA), often employed to combat biotrophic pathogens in vascular plants, modestly accumulates during interactions with necrotrophic fungi in *P. patens* (Ponce de Leon et al. 2012). In addition, genes involved in the synthesis of SA precursor molecules (*PAL*) are induced in pathogen-treated moss (Ponce de Leon et al. 2007, Oliver et al. 2009, Ponce de Leon et al. 2012, Reboledo et al. 2015, Overdijk et al. 2016) and several moss and liverwort species collected from field sites display varying levels of endogenous SA (Drabkova et al. 2015). Together, these studies demonstrate a capacity for SA biosynthesis in bryophytes, although the conservation of SA function during bryophyte–microbe interactions remains to be clarified. In contrast, the wounding and defense-related oxylipin hormone jasmonic acid (JA), which is associated with defenses against herbivores and necrotrophic pathogens, does not appear to play a prominent role during plant–microbe interactions in bryophytes. Both mosses and liverworts are unable to synthesize JA in experimental conditions (Stumpe et al. 2010, Ponce de Leon et al. 2012, Yamamoto et al. 2015) and only trace levels of JA are detected in field-collected mosses and liverworts (Drabkova et al. 2015). Bryophytes appear to utilize the JA precursor molecule 12-oxo-phytodienoic acid (OPDA) rather than JA itself, as OPDA accumulates in wounded liverworts as well as wounded and infected moss (Ponce de Leon et al. 2012, Yamamoto et al. 2015). Moreover, exogenous application of OPDA causes growth inhibition, defense gene activation and ROS accumulation in moss, similar to MAMP-treated plants (Oliver et al. 2009, Ponce de Leon et al. 2012, Ponce de Leon et al. 2015). Interestingly, exogenous methyl-JA (MeJA) treatments cause growth inhibition in moss, while JA has no effect on liverwort growth (Ponce de Leon et al. 2012, Yamamoto et al. 2015). This may suggest that mosses contain aspects of JA signaling that are absent in liverwort lineages, although the effect of MeJA on liverwort growth has not been tested. Given that mosses cannot synthesize JA/MeJA, further experimentation is required to determine if bryophyte MeJA perception arose more recently from interplant communication with MeJA-producing plants. In addition to classic defense-related oxylipins such as OPDA and JA, recent characterization of the αDOX (α-dioxygenase) enzyme in *P. patens* demonstrated that additional oxylipin species accumulate and participate in defense responses to necrotrophic pathogens in moss (Machado et al. 2015). Furthermore, auxin biosynthesis and signaling are activated during defense responses in moss (Reboledo et al. 2015, Mittag et al. 2015, Alvarez et al. 2016). The overaccumulation of free IAA (auxin) in *P. patens gh3* double-knockout lines was associated with increased resistance to *Pythium* infection, demonstrating a protective role for auxin accumulation during defense (Mittag et al. 2015). Taken together, these studies suggest that host–microbe interactions probably contributed to the evolution of complex plant hormone signaling cascades during the expansion of plants onto land.

In addition to locally induced MAMP-triggered responses, a study performed in the moss *Amblystegium serpens* suggests that systemic acquired resistance (SAR)-like responses are conserved in bryophytes. Localized *Pythium* infections and MAMP treatments in *A. serpens* gametophytes provided enhanced resistance to subsequent infections in distal parts of the same gametophyte (Winter et al. 2014). In angiosperm models, the SAR response protects distal leaves from future infection by mobilizing long-distance immune signals that travel through the vasculature from the site of pathogen attack (Fu and Dong 2013). Since bryophytes are non-vascular plants, the SAR-like response observed in *A. serpens* probably requires cell–cell (symplastic), apoplastic (extracellular) or volatile-mediated immune signaling between adjacent tissues. This form of systemic immunity appears to be mechanistically different from the traditional SAR responses in *Arabidopsis thaliana* and *Nicotiana tabacum* that rely on phloem-mediated long-distance signaling. Rather, the SAR-like response in moss appears similar to ‘adjacent’ resistance responses in barley, where infection in one part of a leaf enhances resistance to subsequent infections in distal parts of the same leaf (Colebrook et al. 2012). Surprisingly, adjacent resistance signaling in barley shares significant overlap with SA-mediated SAR in Arabidopsis (Colebrook et al. 2012), while ‘true’ SAR responses occurring in systemic barley leaves are associated with jasmonates and ABA, but not SA (Dey et al. 2014). The confounding nature of adjacent resistance and the SAR pathways of monocots and dicots suggests that plants may have evolved and adapted these responses several times throughout their evolutionary history. Identifying the mobile signals and associated signaling machinery required for SAR-like adjacent resistance in bryophytes may shed light on the nature of systemic immunity in vascular plants, perhaps resolving the debate as to how and why SAR signaling became so complex in angiosperms.

Bryophytes maintain the molecular tool-kit required to support mycorrhizal symbiosis (Delaux 2017). Orthologs of the calcium and calmodulin-dependent protein kinase (CCaMK) DMI3 (DOES NOT MAKE INFECTIONS3) isolated from two liverworts (*Haplomitrium gibbsiae* and *Treubia lacunosa*) and a hornwort (*Phaeoceros laevis*) complement the mycorrhizal defect in the *Medicago truncatula dmi3* mutant (Wang et al. 2010). Moreover, several bryophytes encode core symbiosis signaling genes (*LysM-RLK*, *DMI1*, *DMI2*, *CCaMK* and *IPD3*), GRAS transcription factor genes (*NSP1*, *NSP2*, *RAM1* and *RAD1*) and downstream components important for symbiosis (*RAM2*, *VAPYRIN*, *STR* and *STR2*). Transcriptome analysis of liverworts (*L. cruciata*) colonized by the AM fungus *R. irregularis* demonstrated that many of these genes are up-regulated during symbiosis, including non-orthologous copies of a symbiosis-inducible H^+^-ATPase and phosphate transporters (Delaux et al. 2015). Interestingly, CCaMK orthologs were also identified in algae, of which the *Closterium peracerosum CpCCaMK* ortholog complemented the mycorrhizal defect of the *M. truncatula Mtccamk* mutant (Delaux et al. 2015). Together, these studies demonstrate that early land plants evolved the core molecular machinery required to support symbiotic endophytes. This ability to sense and respond to symbiotic partners is also observed in early divergent microbes. Early divergent fungi encode receptor proteins with a surprisingly high degree of similarity to plant hormone receptors, which are hypothesized to participate in plant sensing to initiate colonization-associated responses (Herivaux et al. 2017). Moreover, the complement of candidate secreted proteins utilized by the AM fungus *R. irregularis* during the colonization of liverwort (*L. cruciata*) thalli overlaps strong with those expressed during the colonization of monocot and dicot roots (Kamel et al. 2017). Plant sensing is also implicated in bryophyte–cyanobacterial symbioses. Nitrogen-starved hornworts (*Anthoceros*) and liverworts (*Blasia*) produce heat-labile chemical signals to attract free-living *N. punctiforme* cyanobacteria (Meeks and Elhai 2002, Meeks 2003, Adams and Duggan 2008). Upon recognizing these signals, cyanobacteria develop motility-associated structures (hormogonia) that enable chemotactic movement towards bryophyte hosts (Adams and Duggan 2008). These studies demonstrate that both plants and fungi have evolved sophisticated means to initiate and sustain beneficial interactions, forming ancient host–microbe alliances that enabled the conquest of terrestrial environments.

### Microbe-derived components impacting bryophyte–microbe interactions

Plant-associated microbes manipulate hosts by secreting proteins and small molecules directly into host cells or into the extracellular spaces (apoplasts) of host tissues. Such molecules are termed effectors, as they have been demonstrated to interact with host machinery to promote colonization (Toruno et al. 2016). Symbiotic and pathogenic bacteria deliver effector molecules into host cells using specialized secretion systems. For instance, the bacterial type 3 secretion system (T3SS), which acts as a molecular hypodermic needle to inject bacterial molecules into host cells, is employed by hemi-biotrophic pathogens and most nitrogen-fixing rhizobacteria alike (Buttner and He 2009, Miwa and Okazaki 2017). Moreover, the bacterial T4SS employed by *Agrobacterium tumefaciens* for the delivery of proteins and bacterial transfer DNA (T-DNA) into host cells is similarly employed by symbiotic rhizobacteria (Lacroix and Citovsky 2016). Filamentous fungi and oomycetes deliver effector molecules using specialized intracellular hyphal structures such as arbuscules and haustoria, which are enveloped by specialized membranes that are differentiated from host plasma membranes (Win et al. 2012, Harrison 2012, Giraldo and Valent 2013, Bozkurt et al. 2015). Extensive evidence suggests that fungal and oomycete haustoria are sites of effector delivery; however, the exact mechanisms involved in effector uptake remain to be clarified (Petre and Kamoun 2014, Lo Presti and Kahmann 2017). In some cases, effectors contain conserved amino acid sequences, such as the RXLR motif of intracellular (cytoplasmic) oomycete effectors, which are hypothesized to act as a signature for uptake into plant cells (Whisson et al. 2007). However, a recent report demonstrated that the RXLR motif of the *P. infestans* Avr3a effector is cleaved during secretion, which suggests a role in secretion rather than uptake into plant cells (Wawra et al. 2017). Moreover, the delivery of cytoplasmic and apoplastic effectors occur using distinct pathways, as the endoplasmic to Golgi trafficking inhibitor BFA (brefeldin A) suppresses the delivery of apoplastic but not cytoplasmic effectors (Giraldo et al. 2013, Wang et al. 2017), which adds further complexity to the nature of filamentous pathogen effector delivery. Similarly to haustoria, intracellular endosymbiotic structures such as arbuscules are believed to be the site of effector delivery, although evidence for effector molecules acting during plant–fungal symbioses is comparatively limited. To date, a small number of candidate mycorrhiza-promoting effectors have been isolated and their precise functions in promoting symbiosis remain to be determined (Fiorilli et al. 2016, Tsuzuki et al. 2016). The SP7 (SECRETED PROTEIN7) effector of *R. irregularis* is the lone exception to this, as it was shown to suppress host immune responses by targeting the ERF19 (ETHYLENE RESPONSE FACTOR19) transcription factor (Kloppholz et al. 2011). Finally, microbes lacking specialized intracellular structures, such as symbiotic ectomycorrhizal fungi and non-haustorial pathogenic fungi such as *Cladosporium fulvum*, must secrete effector proteins into the apoplast and rely on host cell endocytosis for intracellular localization. This is supported by several lines of evidence demonstrating the presence of intracellular effectors secreted by *C. fulvum* (Giraldo and Valent 2013), in addition to the recently characterized MiSSP7 (MYCORRHIZA-INDUCED SMALL SECRETED PROTEIN-7) effector from the ectomycorrhizal fungus *Laccaria bicolor* that acts inside host cells to interfere with JA signaling (Plett et al. 2011, Plett et al. 2014).

**Fig. 2:**
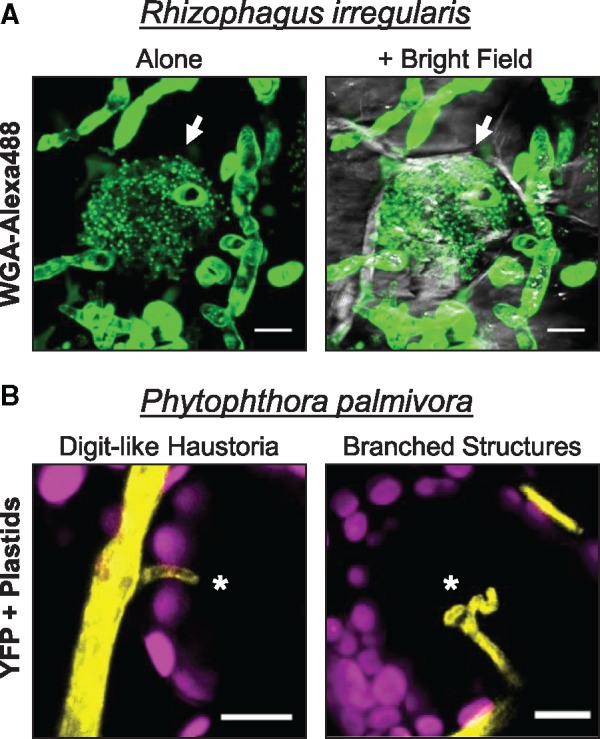
Intracellular biotrophic structures of symbiotic and pathogenic microbes in liverwort cells. (A) Arbusculated liverwort cells. Confocal fluorescence microscopy of sectioned *Lunularia cruciata* thalli stained with WGA (wheat germ agglutinin)–Alexa488 to detect fungal chitin. Plants were grown on vermiculite supplemented with crude *Rhizophagus irregularis* inoculum for 2 months under a long-day photoperiod (16 h light) with attenuated light (approximately 50–70 μE m^−2^ s^−1^). *Z*-stack images display green fluorescence alone or merged with bright field images. Arrows indicate an arbuscule-like structure occupying a cell within the non-photosynthetic storage layer. Scale bars = 10 μm. (B) Digit-like haustoria and branched intracellular structures of the hemi-biotrophic oomycete pathogen *Phytophthora palmivora* deployed in photosynthetic cells of the liverwort *Marchantia polymorpha* (TAK1). Three-week-old TAK1 plants grown on 1/2 MS-B5 (Musharige and Skoog + B5 vitamins) medium(pH 6.5) were inoculated with zoospores of *P. palmivora* (Accession P16830) constitutively expressing endoplasmic reticulum-retained yellow fluorescent protein (YFP). Confocal fluorescence microscopy of TAK1 thalli was performed 3 d after infection. Merged *z*-stack images display YFP fluorescence and plastid autofluorescence (magenta). Scale bars = 10 μm.

Microbial effector proteins converge on similar pathways to manipulate host responses (Win et al. 2012). This is often achieved by interfering with immune signaling, manipulating host secretory pathways, disrupting hormone availability or signaling, controlling host gene expression or diverting host resources to plant–microbe interfaces (Toruno et al. 2016, Bialas et al. 2017). An expanding paradigm suggests that evolutionarily distant microbes have evolved effector proteins that target interconnected hubs of the host cellular machinery. This idea is supported by extensive yeast two-hybrid data that reveal strong overlap in the host machinery of *A. thaliana* targeted by bacterial (*Pseudomonas syringae*), oomycete (*Hyaloperonospora arabidopsidis*) and fungal (*Golovinomyces orontti*) effectors (Mukhtar et al. 2011, Wessling et al. 2014). These results suggests a strong evolutionary pressure for plants to protect vulnerable hubs of host machinery, which probably co-evolved alongside microbial effector proteins during the expansion of plants onto land. To our knowledge, there are no functional studies that assess whether commonly targeted angiosperm hubs are similarly manipulated by effector proteins in early land plant cells. To date, only limited indirect or circumstantial evidence supports the idea that effector proteins manipulate bryophyte hosts. For example, protoplasts derived from the moss *P. patens* as well as sporelings and regenerating thalli of the liverwort *M. polymorpha* are amenable to *Agrobacterium*-mediated genetic transformation, which implies the successful delivery of virulence effectors and T-DNA via the bacterial T4SS into bryophyte cells (Ishizaki et al. 2008, Cove et al. 2009, Kubota et al. 2013). Furthermore, biotrophic intracellular microbial structures such as arbuscules and haustoria are deployed in liverworts (Fig. 2), which supports the idea that effectors may be delivered to liverwort cells. Indeed, comparative transcriptomics analysis revealed the up-regulation of candidate secreted effector proteins employed by *R. irregularis* during mycorrhizal symbiosis in the liverwort *L. cruciata*, which overlapped with the secreted protein repertoire expressed during the colonization of angiosperm roots (Kamel et al. 2017). The hemi-biotrophic oomycete *P. palmivora* similarly up-regulates candidate effector proteins during the colonization of *M. polymorpha* liverwort thalli, including a substantial number of RXLR effectors, small cysteine-rich proteins, cell wall-degrading enzymes and other classes of enzymes typically employed by *Phytophthora* species (Carella et al. 2017). Future efforts to understand how microbial effector proteins and their host targets co-evolved throughout the evolution of land plants will further our understanding of how microbes manipulate plants and may offer predictive insight that could protect crops from emerging diseases.

To overcome effector-triggered susceptibility, plants have evolved highly specialized resistance (R) receptor proteins that recognize cognate effector proteins to induce a robust defense response known as effector-triggered immunity (ETI). This response is generally believed to include a strong and sustained induction of basal defense components and the hypersensitive response (HR), a form of programmed cell death that acts to limit pathogen ingress at the site of infection (Jones and Dangl 2006, Tsuda and Katagiri 2010). The activation of ETI also limits the ability of nitrogen-fixing bacteria to enter into symbiotic relationships with legumes, as effector–R protein compatibilities dictate the host range of different rhizobial species (Yasuda et al. 2016). R protein structure is highly conserved across angiosperms, consisting of a central NB-ARC domain (nucleotide-binding adaptor shared by human APAF-1, plant resistance proteins and CED-4), a C-terminal LRR (leucine-rich repeat) domain and a variable N-terminal CC (coiled-coil) or TIR (Toll/interleukin-1 receptor) domain (Zhang et al. 2017). R protein architecture is conserved in the two bryophyte species surveyed so far, although specific functions for these proteins have not been demonstrated. Preliminary analyses in the liverwort *M. polymorpha* uncovered the presence of typical R genes in addition to a novel class containing an N-terminal α/β-hydrolase domain (Xue et al. 2012). In comparison, the moss *P. patens* encodes typical R genes in addition to those with an N-terminal protein kinase domain (Xue et al. 2012, Tanigaki et al. 2014). How, and if, these candidate R proteins regulate ETI in bryophytes remains to be discovered. The plant α/β-hydrolase domain has been associated with the binding of phytohormones associated with responses to pathogenic and symbiotic microbes such as SA, gibberellic acid, strigolactone and karrikin (Mindrebo et al. 2016). This may suggest a role for α/β-hydrolase domain-containing liverwort R proteins in the detection of effectors that target hormone-binding proteins, or perhaps in the direct monitoring of hormones or microbe-derived phytohormone analogs. The N-terminal kinase domain of moss Kinase-NB-LRR R proteins is perhaps involved in transducing effector-triggered immune signaling

Plants activate immune signaling upon the perception of endogenous damage-associated molecular patterns (DAMPs) that are typical of plant–microbe interactions. DAMPs are liberated during the degradation of host cell walls and cuticles, and are also produced by the proteolytic processing of endogenous pro-hormone polypeptides (Choi and Klessig 2016). Mechanistic studies demonstrating the conservation of DAMP signaling in bryophytes have not been described; however, it is implicated in the responses of moss to elicitors of necrotrophic bacterial pathogens. In several studies, the application of *P. carotovorum* culture filtrate, which is thought to be enriched in cell wall-degrading enzymes and bacterial elicitors, induces moss immunity as demonstrated by the activation of cell wall reinforcement, defense gene expression, programmed cell death and the accumulation of OPDA (Ponce de Leon et al. 2007, Mittag et al. 2015, Alvarez et al. 2016). Moreover, *P. carotovorum* elicitor treatment also activates local auxin signaling and accumulation in *P. patens* (Alvarez et al. 2016), which has been associated with increased resistance to necrotrophic oomycetes in moss (Mittag et al. 2015). Auxin is also associated with biotrophic interactions with symbiotic cyanobacteria in liverworts and hornworts. The colonization of liverwort and hornwort symbiotic cavities induces the formation of multicellular, branched filaments that penetrate cyanobacterial colonies growing within these structures. The activation of these morphological changes is hypothesized to result from cyanobacteria-produced auxin that is secreted into the symbiotic cavity (Adams and Duggan 2008). Given that auxin accumulation protects moss tissues from necrotrophic pathogens and probably influences biotrophy with symbiotic cyanobacteria, it appears that auxin plays a key role for cell survival and growth during plant–microbe interactions in bryophytes. It will be interesting to assess whether auxin influences susceptibility to hemi-biotrophic pathogens in bryophytes as it does in angiosperms (Berens et al. 2017).

## Conclusion and Outlook

The literature discussed in this review demonstrates the diverse range of microbes that interact with bryophytes. Several studies describe conserved host responses to colonization, including cell wall reinforcement, ROS accumulation, phytohormone signaling and defense/symbiosis-related gene expression. Key components of angiosperm symbiosis and defense programs appear to be conserved in bryophytes, which suggests that these programs were first established in early land plant lineages. However, the limited data on bryophyte–microbe interactions have provided only a surface-level understanding of the important evolutionary aspects of symbiosis and immunity in land plants. Further exploration of early diverging land plant lineages is therefore required to address this gap in knowledge, and bryophytes present as ideal candidates given their position as extant descendants of the first plants to have colonized land. Models such as *P. patens* and *M. polymorpha* have already shown promise in molecular plant–microbe research, given their amenability to standard molecular biology techniques and relative ease of genetic manipulation (Ishizaki et al. 2008, Strotbek et al. 2013, Ishizaki et al. 2015). Moreover, genomic resources of key bryophyte species are beginning to become available (Rensing et al. 2008, Szovenyi 2016, Bowman et al. 2017, Delmans et al. 2017), which will facilitate future phylogenomic efforts to understand the evolution of important symbiosis/defense-related host machinery. In addition, the use of bryophyte models may enable future studies aimed at understanding how microbes evolved the capacity for symbiosis or pathogenesis during the colonization of land. Such studies may reveal that (i) weak biotrophic pathogens were domesticated and transitioned into symbionts; (ii) cheating symbionts progressed into stronger and stronger pathogens; or (iii) that both symbiont–pathogen and pathogen–symbiont transitions are possible. At any rate, understanding how pathogens and symbionts colonize early land plant tissues may shed light on this dichotomy, revealing highly conserved host processes required for the establishment of the various plant–microbe interactions observed in nature.

## Funding

The Schornack group is funded by the Gatsby Charitable Foundation [RG62472]; the Royal Society [RG69135]; the BBSRC OpenPlant initiative [BB/L014130/1]; and the Natural Environment Research Council [NERC; NE/N00941X/1].

## Abbreviations

AM: arbuscular mycorrhiza
CCaMK: calcium and calmodulin-dependent protein kinase
DAMP: damage-associated molecular pattern
ETI: effector-triggered immunity
JA: jasmonic acid
LRR: leucine-rich repeat
MAMP: microbe-associated molecular pattern
MTI: MAMP-triggered immunity
OPDA: 12-oxo-phytodienoic acid
R: resistance
ROS: reactive oxygen species
SA: salicylic acid
SAR: systemic acquired resistance
T-DNA: transfer DNA
T3SS: type III secretion system
T4SS: type IV secretion system
